# Sandwiching Sulfur into the Dents Between N, O Co-Doped Graphene Layered Blocks with Strong Physicochemical Confinements for Stable and High-Rate Li–S Batteries

**DOI:** 10.1007/s40820-020-00477-3

**Published:** 2020-07-13

**Authors:** Mengjiao Shi, Su Zhang, Yuting Jiang, Zimu Jiang, Longhai Zhang, Jin Chang, Tong Wei, Zhuangjun Fan

**Affiliations:** 1 Key Laboratory of Superlight Materials and Surface Technology, Ministry of Education, College of Material Science and Chemical Engineering, Harbin Engineering University, Harbin, 150001 People’s Republic of China; 2 Key Laboratory of Energy Materials Chemistry, Ministry of Education, Key Laboratory of Advanced Functional Materials, Autonomous Region, Institute of Applied Chemistry, Xinjiang University, Urumqi, 830046 People’s Republic of China; 3 State Key Laboratory of Heavy Oil Processing, School of Materials Science and Engineering, China University of Petroleum, Qingdao, 266580 People’s Republic of China

**Keywords:** Graphene, Physicochemical confinement, Cycle stability, Shuttle effect, Li–S batteries

## Abstract

The development of lithium–sulfur batteries (LSBs) is restricted by their poor cycle stability and rate performance due to the low conductivity of sulfur and severe shuttle effect. Herein, an N, O co-doped graphene layered block (NOGB) with many dents on the graphene sheets is designed as effective sulfur host for high-performance LSBs. The sulfur platelets are physically confined into the dents and closely contacted with the graphene scaffold, ensuring structural stability and high conductivity. The highly doped N and O atoms can prevent the shuttle effect of sulfur species by strong chemical adsorption. Moreover, the micropores on the graphene sheets enable fast Li^+^ transport through the blocks. As a result, the obtained NOGB/S composite with 76 wt% sulfur content shows a high capacity of 1413 mAh g^−1^ at 0.1 C, good rate performance of 433 mAh g^−1^ at 10 C, and remarkable stability with 526 mAh g^−1^ at after 1000 cycles at 1 C (average decay rate: 0.038% per cycle). Our design provides a comprehensive route for simultaneously improving the conductivity, ion transport kinetics, and preventing the shuttle effect in LSBs.

**Electronic supplementary material:**

The online version of this article (10.1007/s40820-020-00477-3) contains supplementary material, which is available to authorized users.

## Highlights

- N, O co-doped graphene layered block (NOGB) was prepared as sulfur host for lithium–sulfur batteries.
- The NOGB/S shows good rate performance due to robust electrochemical kinetics.
- The strong physicochemical confinement leads to an improved cycling stability.

**Electronic supplementary material:**

The online version of this article (10.1007/s40820-020-00477-3) contains supplementary material, which is available to authorized users.

## Graphical abstract

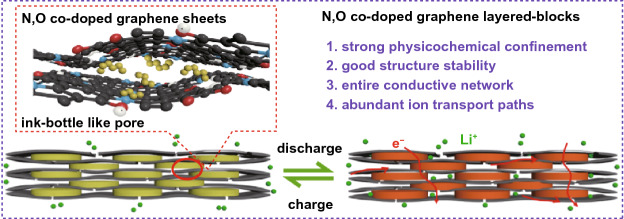

## Introduction

Lithium–sulfur batteries (LSBs) have attracted widespread attention owing to the high theoretical specific capacity (1675 mAh g^−1^), low cost, and natural abundance of sulfur [1, 2]. However, the application of LSBs is severely restricted by two issues: low sulfur utilization due to the sluggish electrochemical kinetics of the non-conductive sulfur; fast capacity decay from the large volume expansion (~ 80%) and the shuttle effect of lithium polysulfides (Li_2_S_*x*_, 4 ≤  *x* ≤ 8) during charge–discharge process [3, 4]. To overcome these problems, various approaches such as developing multifunctional sulfur hosts [2], configuring with modified separators [5–9], and modifying the electrolyte with additives [10–13] have been developed previously [14, 15]. Among them, one of the most promising ways is to confine sulfur into conductive porous carbon scaffolds.

Researchers have made significant efforts on designing conductive carbon hosts such as hollow carbon spheres [16–18], activated carbons [19, 20], carbon nanotubes/fibers [5, 21, 22], and graphene nanosheets [4, 23, 24] for simultaneously improving the conductivity and physical confining sulfur species. However, the physical confinement by carbon hosts is not sufficient to suppress the shuttle effect over long-term cycling because of the weak interaction between the nonpolar carbon hosts and polar polysulfides [25]. Besides, the large volume expansion of sulfur during lithiation also causes the serious dissolution of the polysulfides out of the nanopores. To overcome this issue, researchers proposed a chemisorption method of polysulfides by doping heteroatoms or compounding metal compounds (e.g., MoS_2_ [26, 27], MoSe_2_ [28], TiO_2_ [29–31], and MnO_2_ [32, 33]) in carbon scaffolds. Particularly, nitrogen and/or oxygen doping has been proved to be a simple but effective way for enhancing the chemisorption ability of lithium polysulfides through the formation of strong Li–N/O bonds as well as improving the surface polarity [25, 34–37]. As a result, a promising carbon host could provide large free space for physically confining sulfur species and buffering the volumetric expansion, strong chemisorption of polysulfides for suppressing the shuttle effect, and highly conductive network for improving the electrochemical kinetics.

**Scheme 1:**
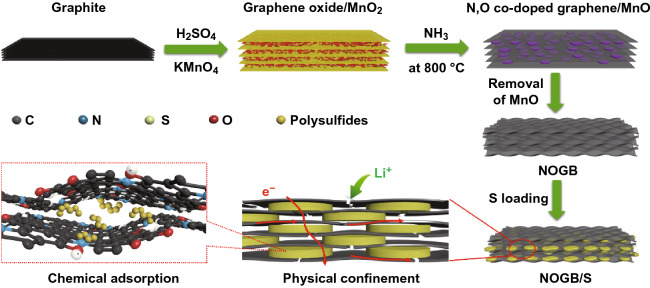
Preparation process of the NOGB/S composite

Herein, we report a rational designed N, O co-doped graphene layered block (NOGB) with many dents on the graphene sheets as sulfur host to improve the stability and rate performance of LSBs, as shown in Scheme 1. The graphene oxide/MnO_2_ nanowires layered stacking composite was first prepared by the modified Hummers’ method yet without removing the MnO_2_. Then, the NOGB was prepared by thermal treatment of the composite in the NH_3_ atmosphere and subsequent removing the resulted MnO nanoplatelets. Due to the MnO nanoplatelets as spacers, there are many dents on the graphene sheets. This unique structure can effectively suppress the shuttle effect of polysulfides by the strong physicochemical confinement. Moreover, the close contact between graphene and sulfur platelets and abundant micropores on the graphene sheets as ion migration channels enable the NOGB/S composite with robust electrochemical kinetics. The NOGB/S with a high sulfur content of 76 wt% shows a high capacity of 1413 mAh g^−1^ at 0.1 C, high rate performance of 433 mAh g^−1^ at 10 C, and remarkable stability with 526 mAh g^−1^ after 1000 cycles at 1 C (average decay rate: 0.038% per cycle), which are among the best of the reported results.

## Experimental Section

### Material Synthesis

#### Synthesis of NOGB

Graphene oxide/MnO_2_ nanowires composite was prepared by the modified Hummers’ method [38]. Typically, 5 g natural graphite powder was added into 115 mL concentrated H_2_SO_4_ at 0 °C under vigorous magnetic stirring for 0.5 h; then, 30 g KMnO_4_ was added and stirring at ~ 38 °C for 2 h. The mixture temperature was adjusted to 70 °C by adding 230 mL deionized (DI) water and stirred for another 2 h to obtain graphene oxide/MnO_2_ slurry. The graphene oxide/MnO_2_ composite was obtained by lyophilization after washing the slurry with DI water to neutral. After that, the graphene oxide/MnO_2_ was treated in an ammonia atmosphere at 800 °C for 2 h with the heating rate of 3 °C min^−1^ to get N, O co-doped graphene/MnO composite. Subsequently, the composite was washed by a mixture of 80 mL 0.1 M H_2_SO_4_ and 10 mL H_2_O_2_ solution (35 wt%) and DI water repeatedly to remove the MnO. Finally, the NOGB was obtained by lyophilization.

For comparison, graphene block (GB) without N doping was prepared by the same process as NOGB except thermal treatment in the N_2_ atmosphere. Reduced graphene oxide (RGO) was prepared by thermal treatment of graphene oxide in N_2_ atmosphere at 800 °C for 2 h with the heating rate of 3 °C min^−1^.

#### Synthesis of NOGB/S Composites

Typically, NOGB was mixed with sublimed sulfur (99.98%, Sinopharm) at a mass ratio of 1:4. The mixture was heated to 155 °C and kept for 12 h in a N_2_ atmosphere. After cooling down to room temperature, the NOGB/S with the sulfur content of 76 wt% was obtained. For comparison, the GB/S and RGO/S composites were also prepared by the same process with both 71 wt% sulfur content.

### Material Characterizations

The structural characterizations were conducted by scanning electronic microscopy (SEM, CamScan Mx2600FE) and transmission electronic microscopy (TEM, JEOL JEM-2010). X-ray diffraction patterns and Raman spectra of the as-prepared samples were measured by a Bruker D8 Discovery X-ray diffractometer and a Jobin–Yvon HR800 Raman spectrometer with 532 nm wavelength incident laser light. X-ray photoelectron spectroscopy (XPS) measurement was taken on a PerkinElmer PHI-5700 ESCA System with monochromated Al Kα radiation (energy 1486.6 eV). Thermogravimetric analysis (TGA, STA 449 F5) was used to measure the mass contents of sulfur in the composites. N_2_ adsorption–desorption isotherm at 77.4 K (Quantachrome IQ2) was characterized the pore structures and specific surface area of the samples using the Barrett–Joyner–Halenda (BJH) method and Brunauer–Emmett–Teller (BET) method, respectively. The microporous structure of NOGB is measured by the CO_2_ adsorption–desorption isotherm at 0 °C.

### Electrochemical Measurements

The standard 2032 coin-type cell was used for measuring the electrochemical performance of the samples. The cathode slurry was prepared by mixing the as-prepared sample (70 wt%), conductive carbon black (20 wt%), and poly(vinylidene fluoride) binder (10 wt%) in an *N*-methyl-2-pyrrolidone solvent. The slurry was then cast onto an aluminum foil, dried at 60 °C for 12 h in a vacuum oven to prepare cathode electrode. The areal sulfur loading of the electrodes was about 1.2 mg cm^−2^. The electrochemical performance of the NOGB/S electrodes with a high sulfur loading of 4.4 mg cm^−2^ was also measured. The electrolyte used was 1 M lithium bis(trifluoromethanesulfonyl) imide (LiTFSI) and 2 wt% lithium nitrate (LiNO_3_) dissolved in 1:1 (v/v) 1,3-dioxolane/1,2-dimethoxyethane (DOL/DME). The electrolyte-to-sulfur ratio for the coin cell assembly was controlled as ca. 20 µL mg^−1^ for the sulfur loading of 1.2 mg cm^−2^ and 12 µL mg^−1^ for the sulfur loading of 4.4 mg cm^−2^ according to the previous works [22, 37]. Then, the cells consisting of the cathode electrode, a Celgard 2300 separator, a lithium metal anode were assembled in an argon-filled glove box. Galvanostatic charge–discharge at different currents was tested in a voltage range of 1.6–2.8 V using a battery test system (Wuhan LAND, CT2100A, China). Cyclic voltammetry (CV) with a scan rate of 0.1 mV s^−1^ between 1.6–2.8 V and electrochemical impedance spectroscopy (EIS) were performed on an electrochemical station (Autolab PGSTAT302N). Fresh batteries were used for the EIS test at the open-circuit potential. The frequency is ranging from 100 kHz to 10 mHz at a voltage amplitude of 5 mV. The capacities were calculated based on the mass of sulfur.

## Results and Discussion

**Fig. 1:**
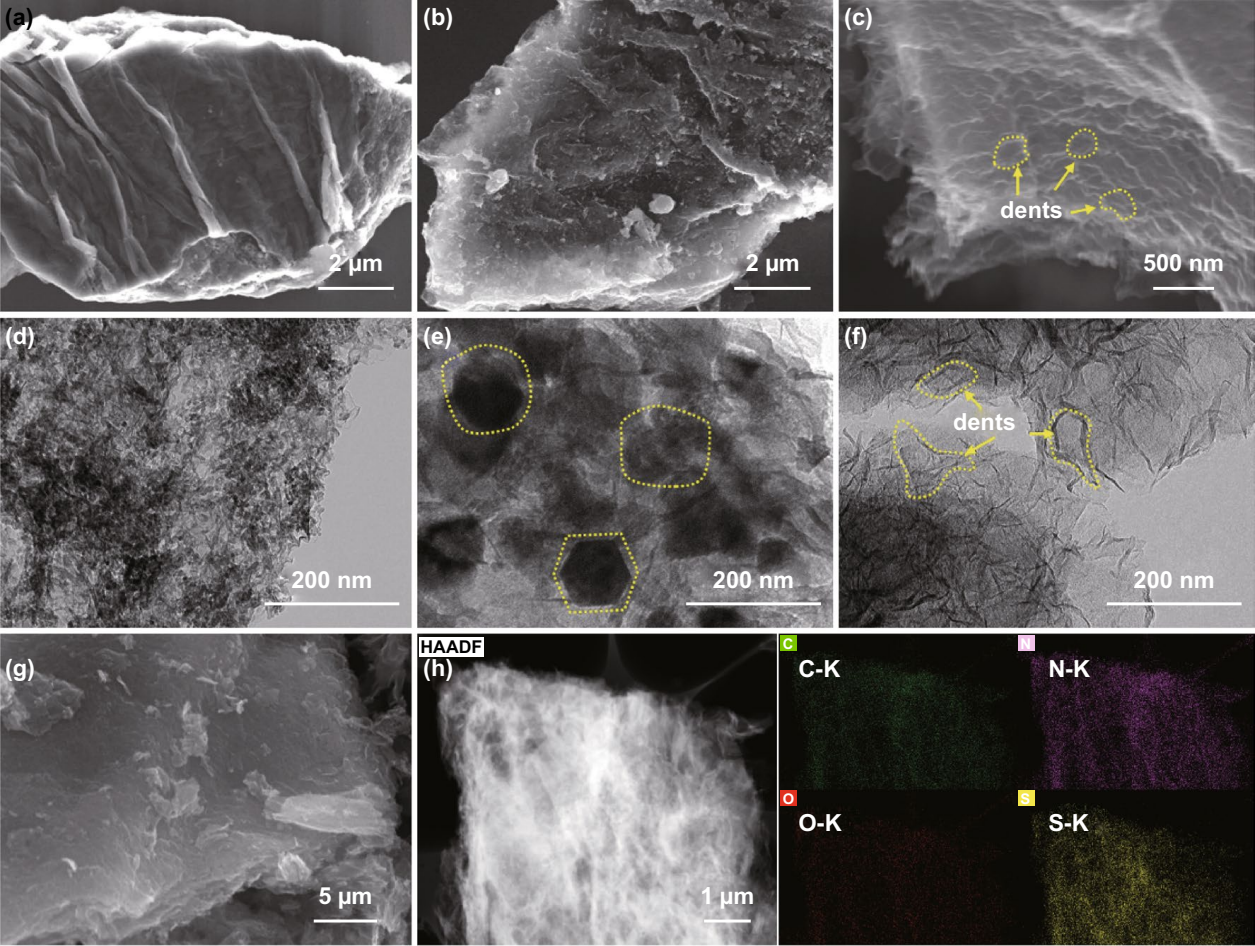
SEM images of **a** graphene oxide/MnO_2_, **b** N, O co-doped graphene/MnO, and **c** NOGB. TEM images of **d** graphene oxide/MnO_2_, **e** N, O co-doped graphene/MnO, and **f** NOGB. **g** SEM images of the NOGB/S. **h** STEM image of the NOGB/S and the corresponding elemental mapping images of carbon, nitrogen, oxygen, and sulfur

Scheme 1 illustrates the preparation process of the NOGB and the NOGB/S composite. KMnO_4_ is intercalated between the graphite layers and then reduced to uniformly distributed MnO_2_ nanowires [38], leading to the formation of a graphene oxide/MnO_2_ sandwich structure (Fig. 1a). Through thermal treatment at 800 °C in NH_3_ atmosphere, the MnO_2_ nanowires are converted to MnO nanoplatelets due to the interlayer confinement, simultaneous leading to the nitrogen doping of the graphene (Figs. 1b, e and S1). After removing the MnO nanoplatelets, the obtained NOGB maintains the block appearance with many dents between the stacking layers (Fig. 1c, f). The sulfur is then loaded into NOGB through melting diffusion, and there are no obvious agglomerated sulfur particles on the surface of NOGB/S (Fig. 1g, h), indicating the full incorporation of sulfur into the dents of the graphene sheets. For comparison, the GB/S with the similar structure as NOGB/S also shows no obvious sulfur particles (Fig. S2), but many large sulfur particles are observed on the surface of RGO/S (Fig. S2). Thus, the RGO with an open structure cannot encapsulate sulfur species effectively. The element distribution of NOGB/S further confirms that N element is homogeneous distribution in the block skeleton, and sulfur is well confined in the NOGB host (Fig. 1h). This indicates the strong capillary absorption of sulfur by the special dent structure of NOGB.

**Fig. 2:**
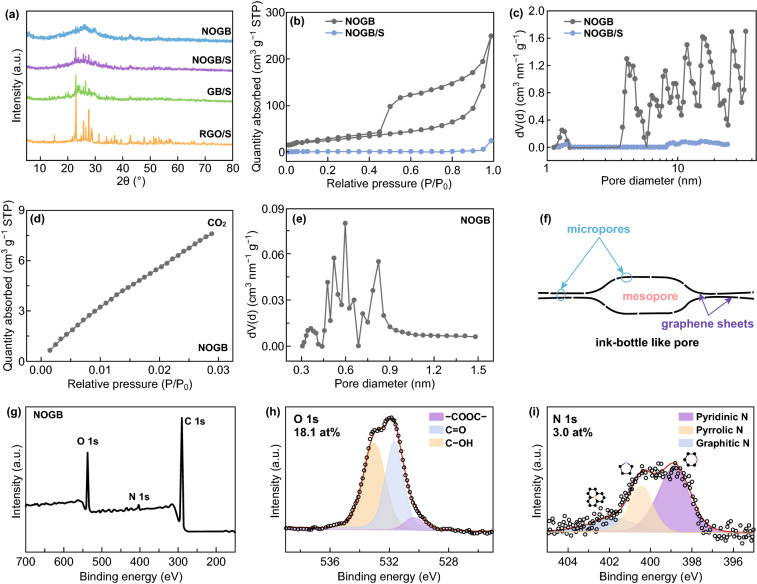
**a** XRD patterns of the NOGB, RGO/S, GB/S, and NOGB/S. **b** N_2_ adsorption–desorption isotherms of the NOGB and NOGB/S and **c** corresponding pore size distribution. **d** CO_2_ adsorption–desorption isotherm of NOGB and **e** corresponding micropore size distribution. **f** Schematic illustration of the porous structure of the NOGB. **g** XPS survey spectrum, **h** O 1s, and **i** N 1s spectra of the NOGB

XRD was performed to investigate the composition and crystalline structure evolution of the products. As shown in Fig. S3, the MnO_2_ in graphene oxide/MnO_2_ show a tetragonal α-MnO_2_ phase (JCPDS No. 04-0141). After calcination at 800 °C, the MnO_2_ is converted to cubic phase MnO in the N, O co-doped graphene/MnO (JCPDS No. 07-0230, Fig. S3). The NOGB, GB, and RGO show the similar dispersive diffraction peak (002) at ~ 25°, indicating their thin layer stacking structure (Figs. 2a and S4) [39]. After impregnating with sulfur, a series of sharp diffraction peaks in the composite materials correspond to the orthorhombic sulfur (JCPDS No. 08-0247). It should be noticed that the diffraction peaks of sulfur in the NOGB/S and GB/S are weaker than those in RGO/S, indicating that the sulfur shows a thin layer structure with low crystallinity due to the confinement in the dents of the graphene blocks (Fig. 1e, h). The contents of sulfur impregnated in NOGB, GB, and RGO obtained by the TG analysis are 76, 71, and 71 wt% (Fig. S5), respectively. In the Raman spectra of NOGB, GB, and RGO (Fig. S6), two peaks at 1358 cm^−1^ (D-band) and 1580 cm^−1^ (G-band) are assigned to the disordered and graphitic regions in graphene, respectively [40]. The NOGB shows the lowest *I*_*D*_/*I*_*G*_ ratio of 0.91 among the samples (GB: 0.93, RGO: 0.97), implying good electrical conductivity.

The specific surface areas and porous structure of the NOGB and NOGB/S are obtained by the N_2_ adsorption–desorption isotherms (Fig. 2b-e and Table S1). The NOGB has a surface area of 92.72 m^2^ g^−1^ with a large pore volume of 0.382 cm^3^ g^−1^. Importantly, it shows a typical IV-type sorption behavior and a H2-type hysteresis loops with an obvious hysteresis loop at the middle and high-relative-pressure region (0.4–1.0), indicating that the dents on graphene sheets with the width of 3–10 nm show an “ink-bottle”-like mesoporous structure (Fig. 2f). This porous structure is much favorable for trapping sulfur species during charge–discharge. After impregnation of sulfur, the surface area and pore volume of the NOGB/S drastically decrease to 2.560 m^2^ g^−1^ and 0.037 cm^3^ g^−1^, revealing that the thin sulfur nanoplatelets are fully contacted with the conductive graphene networks. CO_2_ adsorption–desorption is a powerful method to determine the microporous structure of materials. From the isotherm given in Fig. 2d, e, some micropores with a size distribution of 0.4–0.8 nm are formed on the graphene sheets of NOGB due to the decomposition of functional groups during the heating treatment (Fig. 2f). These micropores can play as Li^+^ migration channels during charge–discharge, enabling high rate performance.

The doped N and O groups in carbon scaffold, especially pyridinic N, are vital for the chemical adsorption of polysulfides during charge–discharge through the strong S_*x*_Li–N/O interactions [41]. The chemical compositions and states of the NOGB, GB, and RGO are investigated by XPS analysis, as shown in Figs. 2g-i and S7. The peaks at around 286, 400, and 532 eV correspond to the C 1s, N 1s, and O 1s for NOGB. The contents of C, N, and O are 78.9, 3.0, and 18.1 at%, respectively. The NOGB shows the highest N and O content than that of GB and RGO (Table S2). The XPS N 1s spectrum of NOGB (Fig. 2i) can be deconvoluted into three peaks corresponding to the pyridinic N (398.1 eV), pyrrolic N (399.8 eV), and graphitic N (401.8 eV). And the O 1s spectrum shows three peaks at 530.6, 531.6, and 533.2 eV assigning to lactones O (–COOC–), carbonyl O (C=O), and hydroxyl O (C–OH), respectively [42]. The contents of N and O are calculated based on XPS results and shown in Table S2. The NOGB possesses abundant polar functional groups such as pyridinic N, pyrrolic N, carbonyl O, and hydroxyl O, which can effectively adsorb polysulfides [42]. Besides, the graphitic N can improve the electrical conductivity of the NOGB [34].

**Fig. 3:**
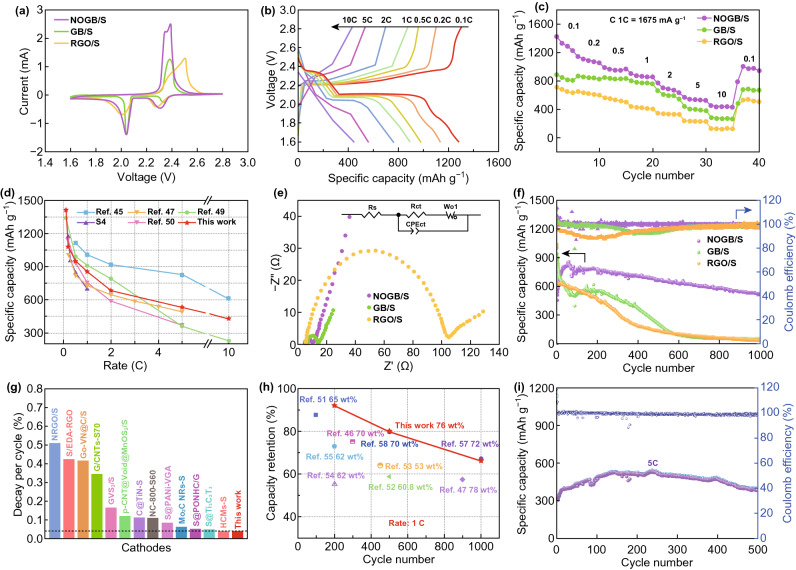
**a** CV curves for the first cycle of NOGB/S, GB/S, and RGO/S. **b** Galvanostatic charge/discharge profiles of the NOGB/S at different rates. **c** Rate capability of the NOGB/S, GB/S, and RGO/S. **d** Rate capacity of the NOGB/S compared with other composites reported in the literature. **e** Nyquist plots of NOGB/S, GB/S, and RGO/S by using fresh cells; the inset is the relevant equivalent circuit model. **f** Comparison of long-term cyclic properties and CE at 1 C. **g** Comparison of the capacity decay rate (per cycle) of the NOGB/S and previously reported materials. (The dashed line corresponds to the decay rate of 0.038%.) **h** Comparison of capacity retention of the NOGB/S and previously reported electrodes at 1C (with the similar S contents in the composites). The comparison was at the same electrolyte system and similar sulfur loadings. **i** Long-term cyclic performance of the NOGB/S at 5 C

Figure 3a presents the first cyclic voltammetry (CV) curves of NOGB/S, GB/S, and RGO/S at the scan rate of 0.1 mV s^−1^. The two reduction peaks at around 2.35 and 2.1 V correspond to the conversion of sulfur to long-chain soluble lithium polysulfides (Li_2_S_*x*_, 4 ≤ *x*≤8), further to short-chain insoluble discharge products (Li_2_S_2_/Li_2_S). The distinct oxidation peak at about 2.4 V is ascribed to the transformation of LiS_2_/Li_2_S_2_ to S_8_ [43]. The sharp shoulder peak at 2.36 V of NOGB/S corresponds to the conversion of LiS_2_/Li_2_S_2_ to Li_2_S_4–8_ and then final to S_8_ [41]. But in GB/S and RGO/S, this shoulder peak is merged to the main peak at 2.38 and 2.50 V, respectively. And the potential difference of the cathode to anode peaks of RGO/S is much higher (0.497 V) than those of the NOGB/S (0.309 V) and GB/S (0.336 V). This indicates the robust electrochemical kinetics for the transformation of LiS_2_/Li_2_S_2_ to S_8_ in NOGB [44]. Furthermore, the CV curves of NOGB/S in the following cycles are almost overlapped when compared with GB/S and RGO/S (Fig. S8), indicating the high chemical reversibility.

The galvanostatic charge–discharge curves of NOGB/S at different rates show typical two plateaus between 1.6 and 2.8 V (Fig. 3b). The unabridged plateaus are still maintained even at a very high rate of 5 C, indicating the remarkable electrochemical kinetics. The corresponding rate performance of NOGB/S was measured at various rates and then back to 0.1 C (Fig. 3c). Obviously, the NOGB/S exhibits high capacities of 1282, 1081, 948, 858, 683, 530, and 433 mAh g^−1^ at 0.1, 0.2, 0.5, 1, 2, 5, and 10 C, respectively. (The capacities are calculated as the average values of the five cycles.) When the current density turns back to 0.1 C, the capacity of NOGB/S restores to a high value of 965 mAh g^−1^. The values are much higher than those of GB/S and RGO/S, and most of the other composite materials reported in the studies (Fig. 3d) [45–48]. The electrochemical kinetics were further studied by EIS (Fig. 3e). The semicircles in the middle frequency region of the Nyquist plots correspond to the charge transfer process, the tail associated with the semi-infinite Warburg diffusion process of Li^+^. The EIS fitting results of *R*_s_ (bulk resistance), *R*_ct_ (charge transfer resistance), and *Z*_W_ (semi-infinite Warburg diffusion resistance) [49] are provided in Table S3. As the sulfur confined in the GB/S and NOGB/S shows a thin layer structure and fully contacts with the conductive graphene frameworks, they show about twenty times lower *R*_ct_ values than the RGO/S with large sulfur particles on the surface. Through N doping, the NOGB/S shows the lowest *R*_ct_ and *Z*_w_ values, and thus the best electrochemical kinetics among the samples.

The cycle performance of the NOGB/S, GB/S, and RGO/S at 1 C was investigated after pre-activation in the first two cycles at 0.05 C (Fig. 3f). Notably, a gradual increase in discharge capacity was observed during the initial first several cycles due to an electrochemical activation process [50], and the NOGB/S reaches its highest capacity of 859 mAh g^−1^ after activation in the first several cycles. It shows an outstanding cycle stability with a very low capacity fading rate of only 0.038% per cycle, maintaining a high-capacity retention of 526 mAh g^−1^ (61.2%). By contrast, the retained capacities of GB/S and RGO/S after 1000 cycles are only 54.9 and 39.1 mAh g^−1^. Moreover, the coulombic efficiency of NOGB/S is 99.8%, also higher than those of GB/S (97.6%) and RGO/S (94.6%), indicating the good reversibility of NOGB/S at high sulfur content. The stability of NOGB/S is better than most of the previously reported electrode materials such as HCMs [47], C@TiN [48], Co-VN@C [46], and some metal oxide-based composites (Fig. 3g, h and Table S4) [46, 47, 51–58]. Notably, the comparison was basically made under the same electrolyte system with same content of LiNO_3_. In addition, when charge–discharge at 5 C, the NOGB/S also shows a large reversible capacity of 477 and 472 mAh g^−1^ after 100 and 400 cycles, respectively. The capacity remains 379 mAh g^−1^ after 500 cycles with a high coulombic efficiency about 98.6% (Fig. 3i). The electrochemical performance of the NOGB hosts prepared at different thermal treatment temperatures was also investigated (Fig. S9). We found that 800 °C was the optimized temperature due to the balance of electrical conductivity and heteroatom content [56, 59, 60]. The NOGB/S electrode with a high sulfur loading of 4.4 mg cm^−2^ was also measured according to the commercialized cathode capacity. An initial discharge capacity of 1373 mAh g^−1^ is achieved at 0.1 C (Fig. S10), leading to an area capacity of 6.0 mAh cm^−2^. The capacity is maintained at 3.3 mAh cm^−2^ after 50 cycles. The good cycle stability clearly demonstrates the advantage of NOGB structure.

**Fig. 4:**
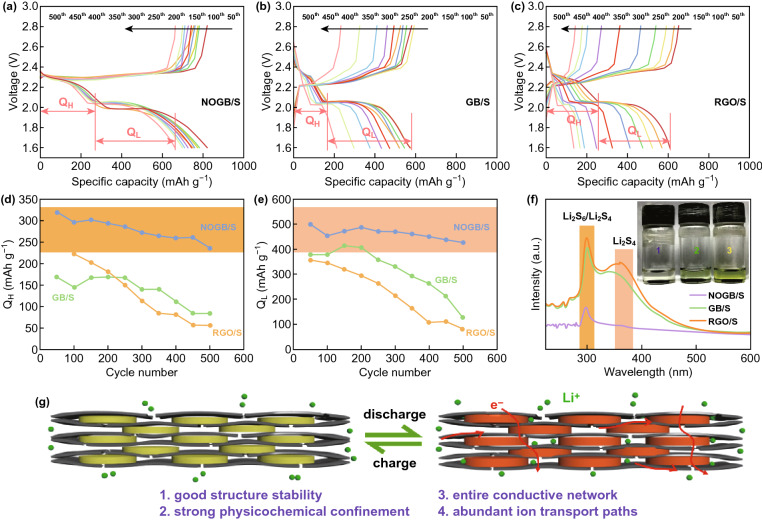
Galvanostatic charge–discharge curves at a rate of 1 C for the **a** NOGB/S, **b** GB/S, and **c** RGO/S. **d** Q_H_ and **e** Q_L_ values through cycling of the three electrodes. **f** UV–Vis absorption spectra collected from the blank electrolyte solutions soaked with (1) NOGB/S, (2) GB/S, and (3) RGO/S electrodes after 50 cycles at 1 C, respectively. The inset photograph shows the color comparison of the solutions containing the corresponding electrodes. **g** Schematic illustration of the NOGB as advanced sulfur host for LSBs

To have an insight into the outstanding performance, the discharge curves at different rates of the samples (Fig. 4a–c) were divided into the high-plateau capacity (*Q*_H_, the adsorption capacity of polysulfide) and low-plateau capacity (*Q*_L_, Li^+^/e^−^ transport in the electrode), as shown in Fig. 4d, e. The soluble Li_2_S_*x*_ (4 ≤ *x*≤8) species are formed when discharging at the high plateau of 2.35 V. The NOGB/S has a high *Q*_H_ value, indicating the suppression of the shuttle effect. On the other hand, the insoluble Li_2_S/Li_2_S_2_ species are generated at discharge low plateau around 2.1 V. The NOGB/S also has a high *Q*_L_ value, indicating the fast conversion ability of soluble polysulfide to insoluble Li_2_S/Li_2_S_2_. Moreover, from the UV–Vis absorption spectra, the two obvious peaks around 300 and 370 nm are attributed to the dissolution of Li_2_S_6_/Li_2_S_4_ and Li_2_S_4_, respectively (Fig. 4f) [58]. The electrolyte containing NOGB/S electrode presents a weaker absorption intensity, and the solution color is nearly close to clean when comparing to the electrolyte containing GB/S or RGO/S electrode. We also disassembled the batteries after 50 cycles and found that the separator for NOGB/S electrode shows a clean surface, demonstrating the strong physicochemical confinement of polysulfides (Fig. S11).

XPS curves of the NOGB/S and GB/S electrodes after discharging to 2.3 V at 0.1 C (formation the long-chain polysulfides, Li_2_S_*x*_, 4 ≤ *x*≤8) were further used to investigate the role of functional groups on the chemical adsorption of polysulfides. A single symmetric peak in GB/S electrode at 55.7 eV is corresponding to the Li–S bond (Fig. S12). The Li 1 s XPS spectrum of NOGB/S electrode shows an asymmetric peak corresponds to the Li–S (55.6 eV) and Li–N (56.3 eV) bonds [47, 61]. The additional peak proves that the nitrogen-bearing functional groups in NOGB have great capability to absorb polysulfides.

The outstanding electrochemical performance of NOGB/S is originated from the unique structure of NOGB host, as illustrated in Fig. 4g. First, the sulfur is sandwiched in the graphene dents and fully attached on the N, O co-doped graphene nanosheets. The unique ink-bottle-like mesopores and highly doped heteroatoms lead to the strong physicochemical confinement of polysulfides during cycling. Second, the good contact between the ultrathin sulfur platelets and graphene scaffold ensures good electrical conductivity. And the small micropores on the graphene sheets promote fast Li^+^ transport in the blocks, leading to the robust electrochemical kinetics. Third, the NOGB host can also buffer the large volume change of sulfur during cycling due to good flexibility of the graphene nanosheets.

## Conclusion

In summary, we have prepared N, O co-doped graphene layered blocks as sulfur host for LSBs through the modified Hummers’ method and subsequent thermal treatment in the NH_3_ atmosphere. The NOGB shows a layered stacking structure with many dents on the graphene sheets, high N and O content, and many micropores. Owing to the unique structure, thin sulfur platelets are strongly confined in the graphene dents, enabling good conductivity, robust electrochemical kinetics, and good structure stability. The NOGB/S shows a high capacity of 1413 mAh g^−1^ at 0.1 C, good rate performance (433 mAh g^−1^ at 10 C), and remarkable long-term cyclic performance. (Average decay rate is 0.038% for 1000 cycles at 1 C.) This strategy may open up an effective route for the design of advanced carbon-based sulfur host materials for achieving high-performance LSBs.

## Electronic Supplementary Material

Below is the link to the electronic supplementary material.

Supplementary material 1 (PDF 1096 kb)
