# Supplementary material for: Sandwiching Sulfur into the Dents Between N, O Co-Doped Graphene Layered Blocks with Strong Physicochemical Confinements for Stable and High-Rate Li–S Batteries
Source: Nanomicro Lett. 2020 Jul 13;12:146. doi: 10.1007/s40820-020-00477-3 (PMC7770931; doi:10.1007/s40820-020-00477-3)
Supplement: Supplementary file 1 — Supplementary material 1 (PDF 1096 kb) [file 40820_2020_477_MOESM1_ESM.pdf]

Supporting Informantion for

Sandwiching Sulfur into the Dents in-between N, O Co-Doped Graphene Layered Blocks with Strong Physicochemical Confinements for Stable and High-Rate Li-S Batteries

Mengjiao Shi[1, #], Su Zhang[2, #], Yuting Jiang[1], Zimu Jiang[1], Longhai Zhang[1], Jin Chang[1], Tong Wei[1, 3, *], Zhuangjun Fan[1, 3, *]

[1]Key Laboratory of Superlight Materials and Surface Technology, Ministry of Education, College of Material Science and Chemical Engineering, Harbin Engineering University, Harbin, 150001, People's Republic of China

[2]Key Laboratory of Energy Materials Chemistry, Ministry of Education; Key Laboratory of Advanced Functional Materials, Autonomous Region; Institute of Applied Chemistry, Xinjiang University, Urumqi 830046, People's Republic of China

[3]State Key Laboratory of Heavy Oil Processing, School of Materials Science and Engineering, China University of Petroleum, Qingdao, 266580, People's Republic of China

[#]Mengjiao Shi and Su Zhang contributed equally to this work

*Corresponding authors. E-mail: weitong666@163.com (T. Wei); fanzhj666@163.com (Z. Fan)

Supplementary Figures and Tables

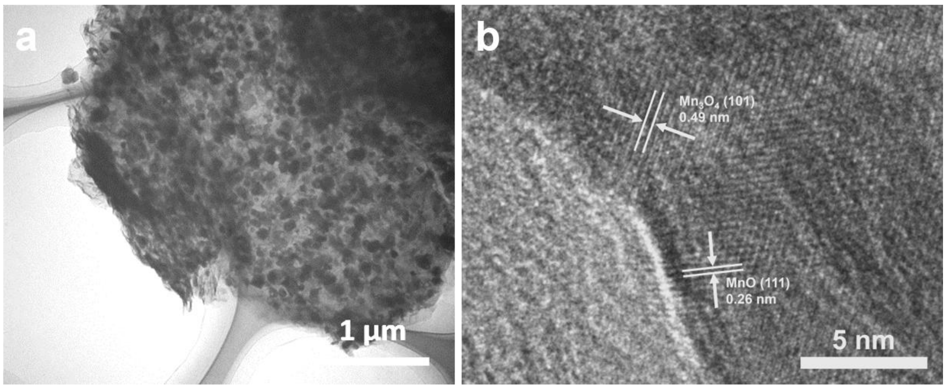

Fig. S1 TEM images of the N, O co-doped graphene/MnO composite

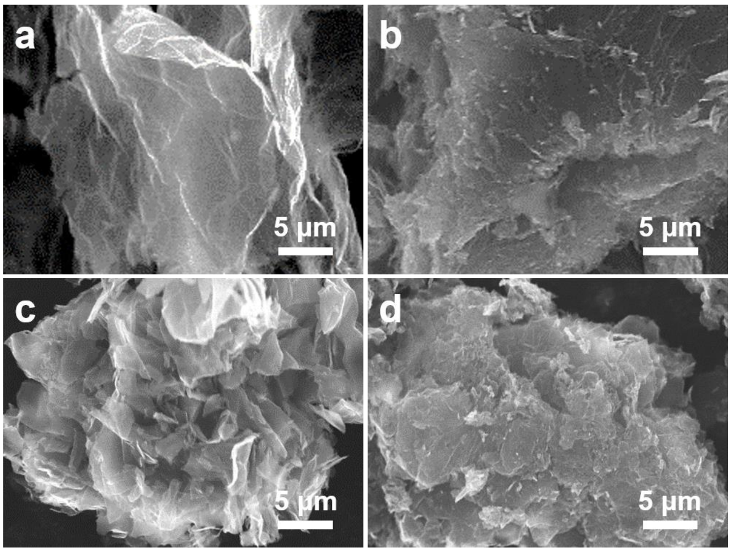

Fig. S2 SEM images of the (a) RGO, (b) GB, (c) RGO/S, and (d) GB/S

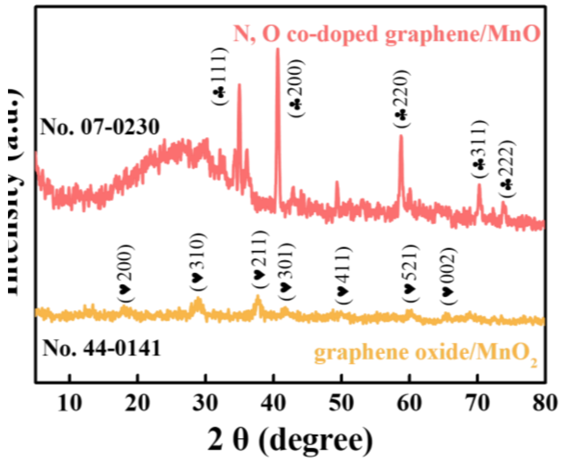

Fig. S3 XRD patterns of the graphene oxide/MnO_2 and N, O co-doped graphene/MnO composites

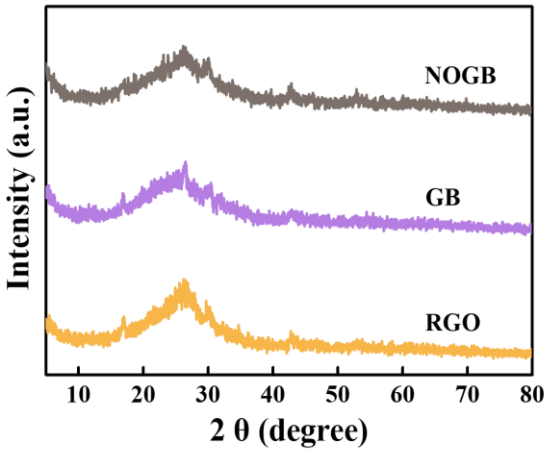

Fig. S4 XRD patterns of the NOGB, GB, and RGO

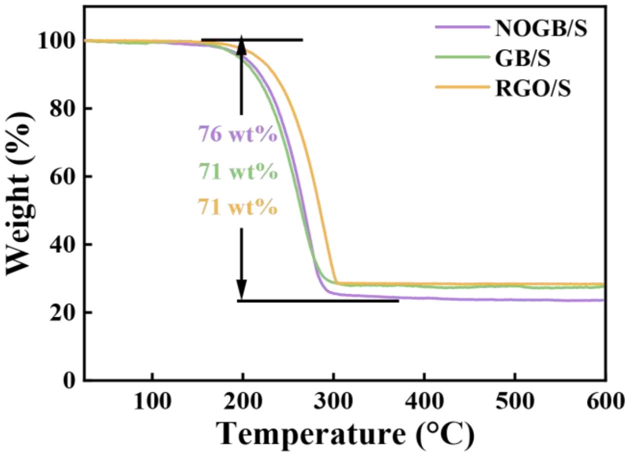

Fig. S5 TGA curves of the NOGB/S, GB/S, and RGO/S

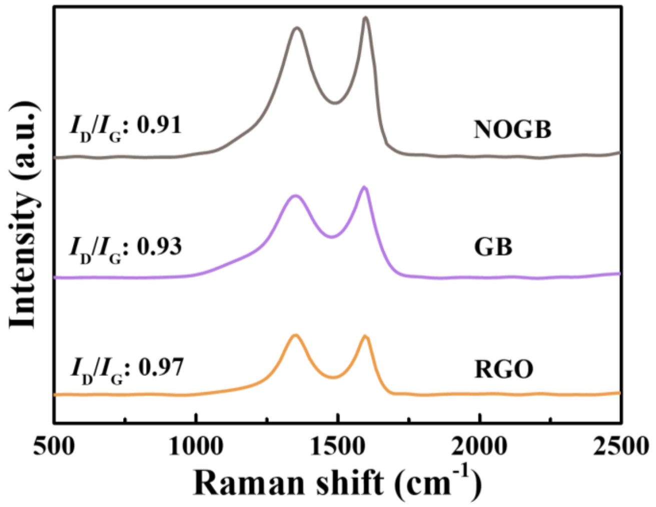

Fig. S6 Raman spectra of the NOGB, GB, and RGO

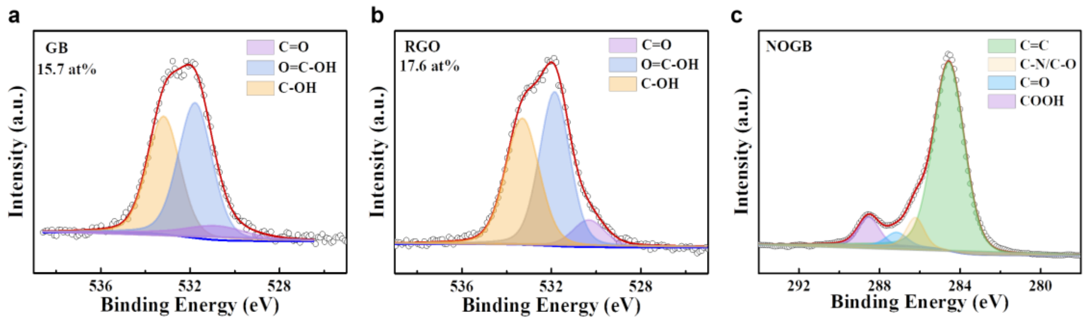

Fig. S7 XPS O 1s spectra of the (a) GB and (b) RGO. (c) XPS C 1s spectrum of the NOGB

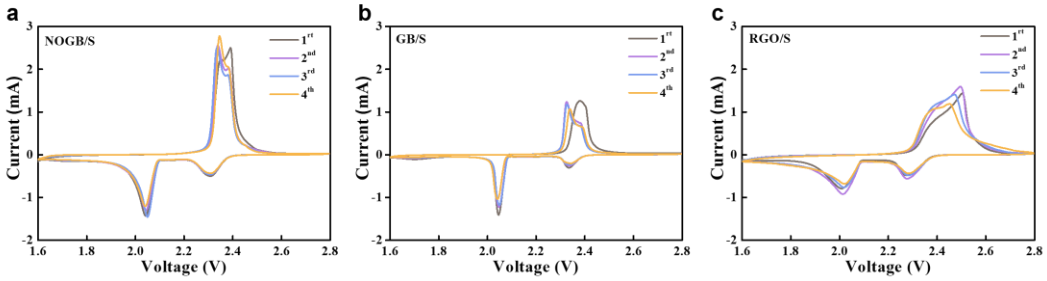

Fig. S8 CV curves for the first four cycles of the (**a**) NOGB/S, (**b**) GB/S, and (**c**) RGO/S

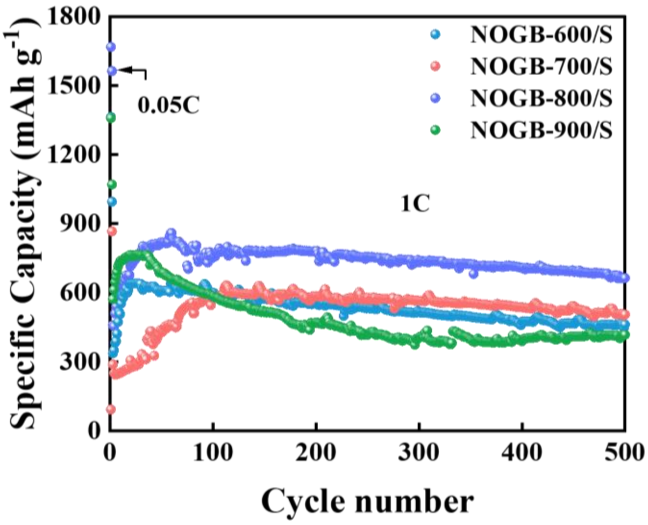

Fig. S9 Comparison of long-term cyclic properties at 1 C of the NOGB/S at different thermal treatment temperature

We have prepared the NOGBs at different thermal treatment temperatures from 600 to 900°C, which were named as NOGB-X (X stands for the temperature). As seen in Fig. S9, the capacity first increases with the thermal treatment temperature, reaching the maximum value for NOGB-800/S, then decreases for NOGB-900/S. This is mainly due to the trade-off between the conductivity and heteroatom content of the doped carbon materials. The thermal treatment temperature is like a double-edged sword. With the increasing of thermal treatment temperature, the electrical conductivity of carbon materials is improved. However, the heteroatom content is drastically decreased due to the unsatisfied thermal stability of the functional groups. Therefore, the electrochemical performance of the carbon host is strongly dependent on the temperature controlled synergy of electrical conductivity, heteroatom doping, and surface polarity.

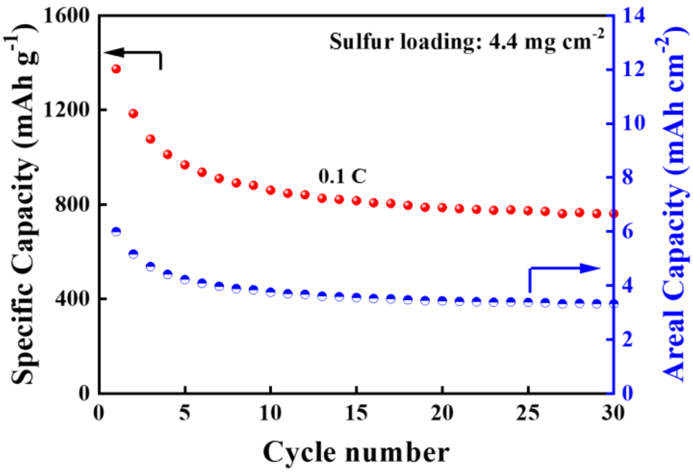

Fig. S10 Gravimetric and areal capacities of the NOGB/S electrode with a sulfur loading of 4.4 mg cm^{-2} at 0.1 C

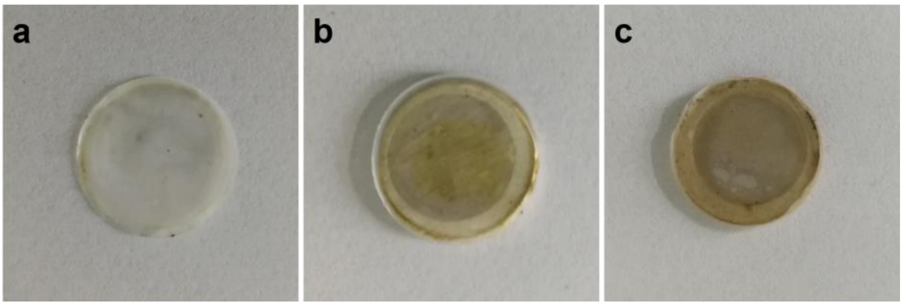

Fig. S11 Photographs of the separators from the disassembled batteries after 50 cycles at 1 C for the (**a**) NOGB/S, (**b**) GB/S, and (**c**) RGO/S electrodes

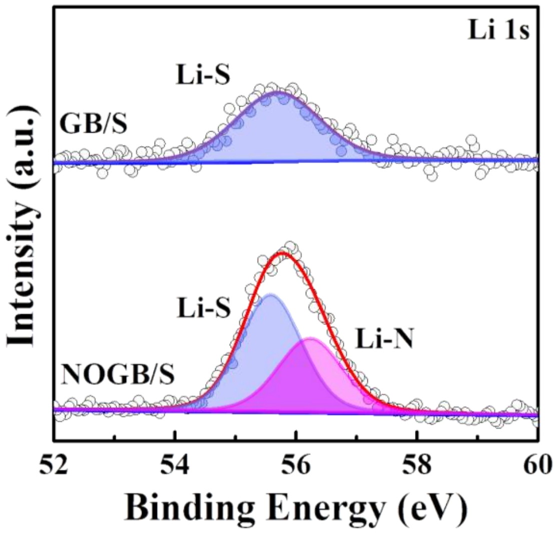

Fig. S12 Li 1s of the GB/S and NOGB/S electrodes after discharging to 2.3 V at 0.1 C

Table S1 Texture properties of the samples measured by N_2 adsorption-desorption isotherms

**Sample**	**S_{BET}[a] (m^2 g^{-1})**	**V_{Total}[b] (cm^3 g^{-1})**
NOGB	92.718	0.382
NOGB/S	2.564	0.037

[a] Specific surface area calculated by BET method

[b] Total pore volume

Table S2 Surface species concentration for the NOGB, GB, and RGO measured by XPS

Sample	C (at%)					N (at%)				O (at%)			
	Total	C=C	C-N/C-O	C=O	COOH	Total	Pyridinic N	Pyrrolic N	Graphite N	Total	-COOC-	C=O	C-OH
NOGB	78.9	81.2%	6.1%	4.6%	8.2%	3.0	55.5%	28.8%	15.7%	18.1	7.1%	43.0%	49.9%
GB	84.3	79.8%	10.7%	1.8%	7.7%	—	—	—	—	15.7	8.9%	49.5%	41.6%
RGO	82.4	80.6%	9.6%	3.8%	6.0%	—	—	—	—	17.6	8.7%	47.6%	43.7%

Table S3 Electrochemical kinetics parameters from the EIS curves for Fig**.** 3e

**Sample**	**R_S** (Ω)	**R_{ct}** (Ω)	**W_R** (Ω)
NOGB/S	4.93	4.08	15.44
GB/S	6.15	6.76	17.80
RGO/S	6.96	80.60	48.79

Table S4 Comparison of the capacities and cycle stability in previous reports

**Sulfur host**	**sulfur content (wt%)**	**sulfur loading (mg cm^{-2})**	**Cyclability**				**Rate Capability**		**Refs.**
			**Cycle No.**	**Rate (C)**	**Capacity (mAh g^{-1})**	**Capacity decay rate (%)**	**Rate (C)**	**Capacity (mAh g^{-1})**	
p-CNT@Void@MnO_2/S	65	0.65–1.06	100	1	526	0.122	2	~450	[S1]
NC-800-S60	60	0.8–1.0	400	0.48	511	0.110	0.96	385	[S2]
Co-VN@C/S	70	~1.3−1.5	300	1	602	0.083	5	490	[S3]
S@PONHC/G	70	1.0−1.2	500	1	607.7	0.052	3	533	[S4]
HCMs-S	78	~1.5	900	1	520	0.04	10	270	[S5]
3DG@NPC/S	70	~1.5	500	1	667	0.083	3	726	[S6]
Our work	76	~1.2	1000	1	526.4	0.038	10	432.7	
			400	5	472.3	0.027			

Supplementary References

[S1] Q. Liu, J. Zhang, S.A. He, R. Zou, C. Xu et al., Stabilizing lithium-sulfur batteries through control of sulfur aggregation and polysulfide dissolution. Small e1703816 (2018). https://doi.org/10.1002/smll.201703816

[S2] J. Zhang, M. Huang, B. Xi, K. Mi, A. Yuan, S. Xiong, Systematic study of effect on enhancing specific capacity and electrochemical behaviors of lithium-sulfur batteries. Adv. Energy Mater. **8**(2), 1701330 (2018). https://doi.org/10.1002/aenm.201701330

[S3] W. Ren, L. Xu, L. Zhu, X. Wang, X. Ma, D. Wang, Cobalt-doped vanadium nitride yolk-shell nanospheres @ carbon with physical and chemical synergistic effect for advanced Li-S batteries. ACS Appl. Mater. Interfaces (2018). https://doi.org/10.1021/acsami.7b18955

[S4] J. Lee, J. Oh, Y. Jeon, Y. Piao. Multi-heteroatom-doped hollow carbon attached

on graphene using lifepo4 nanoparticles as hard templates for high-performance lithium-sulfur batteries. ACS Appl. Mater. Interfaces **10**(31), 26485-26493 (2018). https://doi.org/10.1021/acsami.8b00925

[S5] X. Zhou, J. Tian, Q. Wu, J. Hu, C. Li. N/O dual-doped hollow carbon microspheres constructed by holey nanosheet shells as large-grain cathode host for high loading Li-S batteries. Energy Storage Mater. (2019). https://doi.org/10.1016/j.ensm.2019.06.009

[S6] D. Cheng, Y. Zhao, T. An, X. Wang, H. Zhou, T. Fan, 3D interconnected crumpled porous carbon sheets modified with high-level nitrogen doping for high performance lithium sulfur batteries. Carbon **154** 58-66 (2019). https://doi.org/10.1016/j.carbon.2019.07.094
